# pVAX-iNOS真核表达载体的构建及其抗肺癌作用研究

**DOI:** 10.3779/j.issn.1009-3419.2012.05.02

**Published:** 2012-05-20

**Authors:** 苏娟 叶, 蔚菡 杨, 宇 王, 文婧 欧, 清平 马, 文 朱

**Affiliations:** 610041 成都，四川大学华西医院生物治疗国家重点实验室 State Key Laboratory of Biotherapy and Cancer Center, West China Hospital, Sichuan University, Chengdu 610041, China

**Keywords:** *iNOS*基因, 肺肿瘤, A549细胞株, 基因治疗, 真核表达载体, *iNOS* gene, Lung neoplasms, A549 cell line, Gene therapy, Eukaryotic expression vector

## Abstract

**背景与目的:**

iNOS与NO介导的抗肿瘤效应有关。本研究旨在构建pVAX-iNOS载体并转染A549肺癌细胞，检测其基因的表达并初步探讨*iNOS*基因表达增高后对A549肺癌细胞的抗肿瘤作用。

**方法:**

应用RT-PCR方法扩增人iNOS编码序列的CDS片段，构建pVAX-iNOS载体后转染肺癌A549细胞，通过RT-PCR和Western blot方法检测目的基因的表达；采用MTT法、Hoechst 3235染色和划痕实验分别检测iNOS高表达在体外对肺癌A549细胞增殖、凋亡和迁移作用的影响。

**结果:**

真核表达质粒载体pVAX-iNOS构建成功，iNOS蛋白在转染后的A549细胞中表达升高。pVAX-iNOS转染A549肺癌细胞后能明显诱导细胞发生凋亡并抑制肿瘤细胞的生长和迁移。

**结论:**

本研究成功构建pVAX-iNOS真核表达质粒，高表达iNOS能明显抑制A549细胞的增殖、迁移并促进细胞发生凋亡。本研究有望为临床治疗肺癌提供一个新的有效策略。

## Abstract (en)

**Background and objective:**

The *iNOS* gene is associated with NO-mediated antitumor effects. The aims of this study are to construct a eukaryotic expression plasmid that carries the *iNOS* gene and to detect the expression levels and antitumor effects of the *iNOS* gene on A549 lung cancer cells.

**Methods:**

A DNA fragment of the human iNOS coding sequence was amplified using reverse transcription polymerase chain reaction (RT-PCR). The DNA fragment was subsequently cloned into the multiple cloning sites of the eukaryotic expression vector pVAX. The recombinant plasmid was confirmed using restriction enzyme treatment, PCR, and sequencing and was then transfected into A549 lung cancer cells. The expression of the *iNOS* gene in the A549 lung cancer cells after transfection was verified by RT-PCR and Western blot analysis. The effects of iNOS on cell apoptosis, proliferation, and migration were identified by staining with Hoechst 3235, an MTT assay, and a scratch assay, respectively.

**Results:**

The results of the restriction enzyme digestion, PCR, and sequencing verified the successful construction of the eukaryotic expression plasmid pVAX-iNOS. The *iNOS* gene expression level was increased in the transfected A549 cells. Further experiments also showed increased cell apoptosis among the A549 lung cancer cells transfected with pVAX-iNOS. Meanwhile, the proliferation and migration of A549 cells were significantly inhibited by the enhanced *iNOS* gene expression.

**Conclusion:**

The recombinant eukaryotic expression vector pVAX-iNOS was successfully constructed and transfected into A549 cells. The enhanced *iNOS* gene expression significantly promoted cell apoptosis, whereas the proliferation and migration of A549 cells were inhibited. These findings contribute to the development of novel and effective gene therapies for lung cancer.

肺癌在全世界范围内是发病率和死亡率最高的恶性肿瘤。常规的放化疗和手术治疗是肺癌的基本治疗方法，但大部分患者在确诊时已经发生远处转移，常规治疗手段很难取得较好的临床治疗效果。因此，寻找新的治疗方法非常重要。而肺癌是一个多基因异常导致的疾病。目前基因治疗已被认为是一种极具前景的肿瘤治疗方法。

一氧化氮（nitric oxide, NO）作为一种多功能的小分子物质和人体内重要的信号传递因子之一，是目前国内外肿瘤领域最新研究热点^[1, 2]^。诱导型一氧化氮合酶（inducible nitric oxide synthase, iNOS）是内源性NO合成的关键酶^[3]^。*iNOS*基因在生理状态下不表达或仅少量表达，而在细胞因子等刺激下诱导型表达并产生高水平NO^[4]^。在肿瘤研究^[5-12]^中，高浓度的NO可通过细胞毒作用减缓肿瘤的生长和转移、抑制血管生成、促进分化和凋亡而起到一种抗肿瘤效应。因此，倘若能通过提供NO供体药物或基因转染的方法增加*iNOS*基因的表达并诱导生成高浓度的NO对恶性肿瘤治疗来说将是一个潜在的优势^[13]^。目前有研究^[14-20]^报道，将*iNOS*基因转染到乳腺癌、结直肠癌、前列腺癌、卵巢癌、黑色素瘤、肾癌、甲状腺瘤等肿瘤后均表现出一种强烈的抗肿瘤效应。然而，至今关于*iNOS*基因在肺癌中的基因治疗还没有研究报道。关于*iNOS*基因在肺癌细胞和组织中的表达水平，研究^[5, 21, 22]^报道其总体趋于一种中等量表达的关系，产生的低浓度NO可以促进肿瘤细胞的生长，而高水平的NOS是非小细胞肺癌患者预后的一个有利的因素。因此，通过诱导肺癌细胞*iNOS*基因表达从而激发肺癌细胞释放大量NO可能是抗肺癌治疗的一个新的方法。

本研究拟构建pVAX-iNOS真核表达质粒载体，初步探讨转染A549细胞后iNOS在mRNA和蛋白表达水平的变化及诱导肺癌细胞发生凋亡及抑制细胞增殖和迁移的作用。本研究将为肺癌基因治疗奠定基础，是临床肺癌基因治疗的一个新的探索。

## 1 材料和方法

### 1.1 材料

人iNOS质粒iNOS-pCR4-TOPO购自美国Open Biosystems公司。真核表达质粒载体pVAX购自美国Invitrogen公司。人肺腺癌细胞株A549购自美国ATCC（American Type Culture Collection）。大肠杆菌JM109购自美国Clontech公司，由本实验保种。四氢生物蝶呤（BH4）购于美国Sigma公司。T4 DNA连接酶、*Hind*Ⅲ和*Xba*Ⅰ限制性内切酶购自美国Fermentas公司；PCR Kit、胶回收试剂盒购自日本Takara公司；质粒提取试剂盒购自德国Qiagen公司；Trizol试剂盒和Lipofectamine^TM^ 2000转染试剂购自美国Invitrogen公司；Hoechst 3235购于美国Sigma公司；Anti-iNOS购自Abcam公司；HRP标记二抗购自北京中杉金桥生物技术有限公司；化学发光检测试剂盒购自天根生物技术有限公司；BCA蛋白定量试剂盒购自美国Thermo公司；PVDF膜（0.22 μm）购自Millipore公司；其它试剂均为国产分析纯试剂。6孔板购自美国Coster公司。

### 1.2 方法

#### 1.2.1 pVAX-iNOS真核表达质粒的构建

本实验将购买的iNOS-pCR4-TOPO菌株直接进行划平板、次日挑取单克隆并进行进一步扩大培养，将培养的菌液进行小量质粒提取。然后以iNOS-pCR4-TOPO质粒为模板，采用RT-PCR的方法，扩增iNOS基因的编码区序列并重新连接到pVAX载体上，构建含卡那霉素抗性的真核表达质粒pVAX-iNOS。以iNOS在GenBank中的序列BC130283.1为模板，利用Primer Premier 5.0软件设计引物：上游引物：5'-CGATGGAAAGCTTGCTGTAGAGATGGCCTGTCCTTGGAAATTTCTGT-3'（酶切位点*Hind*Ⅲ）；下游引物：5'-GACGAGGTCTAGATGCCGGCAGCTTTAACCCCTCCTGTAG-3'（酶切位点*Xba*Ⅰ）。PCR产物长度为3, 528 bp。高保真PCR反应体系按试剂盒说明书进行。PCR扩增条件为：94 ℃变性2 min后进入25个PCR循环（94 ℃、30 s，62 ℃、30 s，72 ℃、3.5 min），72 ℃、10 min，4 ℃保存。扩增全长*iNOS*基因序列后，1%琼脂糖电泳鉴定PCR产物，载体和PCR产物经限制性内切酶*Hind*Ⅲ、*Xba*Ⅰ酶切纯化后，将iNOS定向插入pVAX的多克隆位点，转化JM109感受态细胞以卡那霉素筛选阳性克隆，经酶切鉴定正确的克隆由上海Invitrogen公司进行测序。

#### 1.2.2 pVAX-iNOS真核表达质粒的细胞转染

按照Lipofectamine 2000说明书进行操作。本实验均分3组：空白组、pVAX组和pVAX-iNOS组。取对数生长期A549细胞接种于6孔板或96孔板，当细胞融合率达到60%时，将pVAX、pVAX-iNOS质粒分别与Lipofectamine 2000在室温下共同孵育20 min后进行瞬时转染，未转染的A549细胞及转染空载体pVAX的细胞作为阴性对照。次日给予1×10^-5^ mol/L BH4^[16]^（下述所有实验瞬时转染次日均给予相同浓度BH4处理）。转染48 h后分别收集细胞进行后续实验。

#### 1.2.3 RT-PCR检测iNOS在mRNA水平的表达

以Lipofectamine 2000介导pVAX、pVAX-iNOS质粒分别转染A549细胞，方法同上，以未转染的A549细胞和转染空载体pVAX的细胞为阴性对照。48 h后收取细胞用Trizol法提取细胞总RNA，RT-PCR参阅试剂盒说明书进行。利用Primer Premier 5.0软件设计iNOS检测引物序列：上游引物：5'-CCTGAGCTCTTCGAAATCCCACCTGAC-3'，下游引物：5'-AAACTATGGAGCACAGCAATG-3'，PCR产物332 bp。反应条件：94 ℃变性5 min后进入30个PCR循环（94 ℃、30 s，58 ℃、45 s，72 ℃、45 s），4 ℃保存。β-actin：上游引物：5'-CTTAGTTGCGTTACACCCTTTCTTG-3'，下游引物：5'-CTTAGTTGCGTTACACCCTTTCTTG-3'，产物长度：135 bp。反应条件：94 ℃变性3 min后进入35个PCR循环（94 ℃、30 s，57.4 ℃、45 s，72 ℃、1 min），4 ℃保存。1%琼脂糖电泳鉴定PCR扩增产物。

#### 1.2.4 Western blot检测iNOS蛋白表达水平

以Lipofectamine 2000介导pVAX、pVAX-iNOS质粒转染A549细胞，方法同上，以未转染的A549细胞和转染空载体pVAX的细胞为阴性对照。48 h后收取细胞制备蛋白样品，以BCA法进行蛋白定量。将等质量的蛋白进行15%聚丙烯酰胺凝胶电泳分离，电转PVDF膜，电压为86 V，时间90 min。5%脱脂奶粉4 ℃封闭2 h，TBST洗膜后，分别加一抗（兔抗人iNOS单抗和鼠抗人β-actin多抗，均为1:500），4 ℃孵育过夜，TBST洗膜3次；加HRP标记二抗（兔抗鼠IgG 1:5, 000），37 ℃孵育1 h，TBST洗膜3次，等量的ECL反应液A和B混合后加至膜上，显影。

#### 1.2.5 pVAX-iNOS真核表达质粒转染对A549细胞生长的影响

以Lipofectamine 2000介导pVAX、pVAX-iNOS质粒转染A549细胞，方法同上，以未转染的A549细胞和转染空载体pVAX的细胞为阴性对照。转染24 h、48 h和72 h后加入MTT工作液（5 mg/mL PBS配制）20 μL/孔，37 ℃ 5%CO_2_培养箱孵育3 h，弃上清加入150 μL DMSO室温振荡混匀后测*A*_490_吸光值。每个浓度梯度做3个复孔，实验重复3次。

#### 1.2.6 pVAX-iNOS真核表达质粒转染对A549细胞凋亡的影响

以Lipofectamine 2000介导pVAX、pVAX-iNOS质粒转染A549细胞，方法同上，以未转染的A549细胞和转染空载体pVAX的细胞为阴性对照。48 h后加入5 mL新鲜配制的卡诺氏固定液（甲醇:冰乙酸=1:3，现用现配），静置10 min-15 min，弃去固定液，再加入卡诺氏固定液，静置10 min-15 min，重复1次。弃去固定液后于空气中干燥5 min。加入1 mL 0.5 μg/mL的Hoechst 3235避光染色30 min，PBS清洗2次，每次5 min，荧光显微镜下观察凋亡细胞的形态（是否有凋亡小体形成）。

#### 1.2.7 pVAX-iNOS真核表达质粒转染对A549细胞迁移能力的影响

在6孔板中以Lipofectamine 2000介导pVAX、pVAX-iNOS质粒转染A549细胞，方法同上，以未转染的A549细胞和转染空载体pVAX的细胞为阴性对照。24 h后，在各组单层细胞中间划出一道裸露无细胞带，PBS洗涤3次后加入1%血清的1640培养基培养。划痕0 h、6 h、12 h、24 h、36 h和48 h后，取宽度相等的位置观察细胞迁移的情况，于倒置显微镜下观察照相。

### 1.3 统计学分析

采用SPSS 13.0进行统计学分析，结果以Mean±SD表示，以*P* < 0.05为差异具有统计学意义。

## 2 结果

### 2.1 真核表达质粒载体pVAX-iNOS的构建、鉴定和测序

以iNOS-pCR4-TOPO为模板，通过高保真RT-PCR扩增iNOS目的片段，PCR产物经琼脂糖凝胶电泳结果表明，iNOS片段大小正确（大小为3, 528 bp）。将该iNOS PCR产物片段和pVAX载体直接进行*Hind*Ⅲ和*Xba*Ⅰ双酶切。iNOS经*Hind*Ⅲ和*Xba*Ⅰ酶切后约为3, 512 bp。pVAX载体经*Hind*Ⅲ和*Xba*Ⅰ酶切后由环状结构（大小为3, 000 bp）变为2, 920 bp的单链结构。然后将两酶切产物纯化后经T4连接酶进行连接，最后得到真核表达质粒载体pVAX-iNOS（大小为6, 432 bp）。经初步电泳筛选正确后，将构建的pVAX-iNOS质粒载体进行PCR和双酶切鉴定。PCR产物经琼脂糖凝胶电泳结果显示，iNOS片段大小约为3, 528 bp；双酶切产物经琼脂糖凝胶电泳结果显示，pVAX-iNOS质粒载体经*Hind*Ⅲ和*Xba*Ⅰ双酶切后得到3, 512 bp和2, 920 bp左右两条带，与预期结果一致（图 1）。阳性克隆测序结果显示真核表达质粒载体pVAX-iNOS构建成功。

**1:**
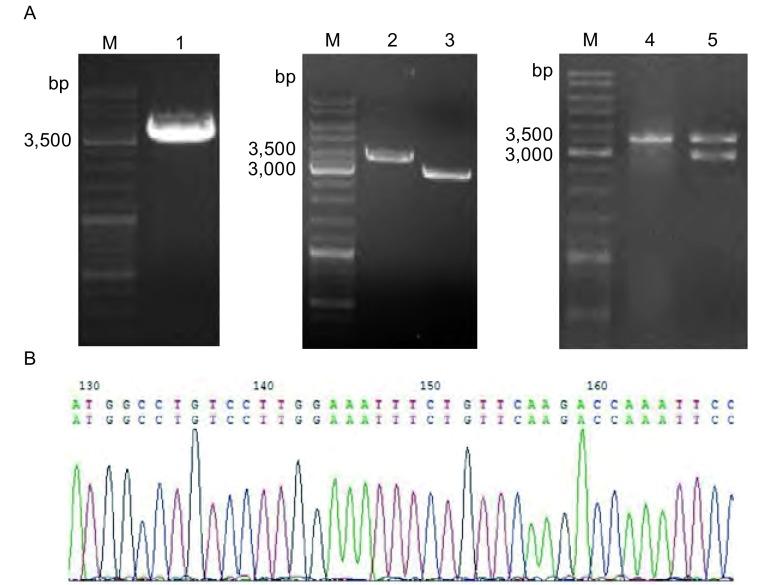
真核表达质粒pVAX-iNOS的构建。A：质粒pVAX-iNOS的构建。M：Marker（DL2000）；1：iNOS PCR产物；2：iNOS PCR产物被*Hind*Ⅲ和*Xba*Ⅰ进行切割并纯化；3：载体pVAX被*Hind*Ⅲ和*Xba*Ⅰ进行切割并纯化；4：阳性克隆的PCR鉴定；5：阳性克隆的双酶切鉴定；B：阳性克隆的测序鉴定（因序列过长，此处只显示以起始码开始的部分测序结果） Construction of eukaryotic expression plasmid pVAX-iNOS. A:Construction of pVAX-iNOS plasmid. M: Marker (DL2000); 1: PCR products of iNOS; 2: The digested and purified PCR products; 3: The digested and purified pVAX; 4: The positive clones confirmed by PCR; 5: The positive clones confirmed by double restriction enzyme digestion; B: Sequencing analysis of the positive clones (part of the sequence).

### 2.2 真核表达质粒转染后A549细胞中iNOS的表达检测

以Lipofectamine 2000介导pVAX、pVAX-iNOS质粒分别转染A549细胞，以未转染的A549细胞及转染空载pVAX的细胞为阴性对照。48 h后分别提取各处理组A549细胞的细胞总RNA，并逆转录为cDNA，RT-PCR检测结果显示iNOS转染后在A549细胞中表达升高（条带大小为332 bp）；相同转染方法处理A549细胞48 h后收取细胞总蛋白，Western blot检测结果显示iNOS在转染pVAX-iNOS后的A549细胞中表达上调，相对分子质量约为135 kDa，与预期大小相符（图 2）。

**2:**
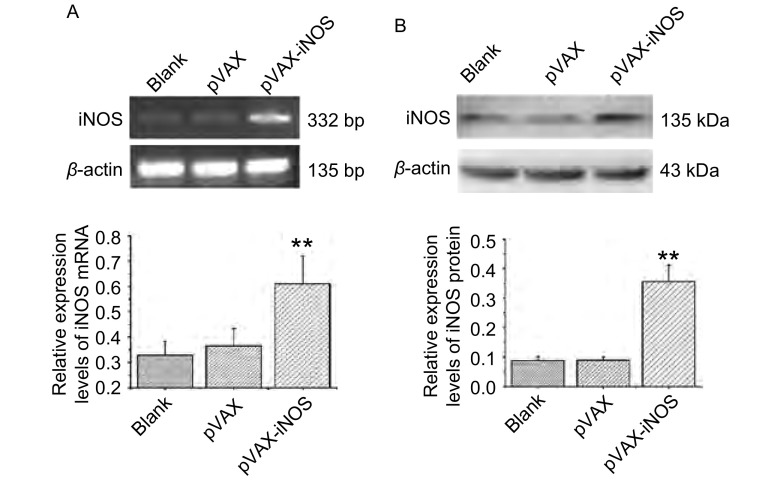
RT-PCR和Western blot检测A549细胞转染pVAX-iNOS 48 h后*iNOS*基因的mRNA和蛋白表达水平及其灰度值分析（^**^P < 0.01）。A：RT-PCR检测空白组、pVAX和pVAX-iNOS转染组A549细胞中iNOS的mRNA表达水平及灰度值分析；B：Western blot检测空白组、pVAX和pVAX-iNOS转染组A549细胞中iNOS的蛋白表达水平及灰度值分析。 The mRNA and protein expression levels of iNOS in A549 cells transfected with pVAX-iNOS for 48 h by RT-PCR and Western blot analysis (^**^P < 0.01). A: The mRNA expression level of iNOS in A549 cells with different treatments by RT-PCR analysis; B: The protein expression level of iNOS in A549 cells with different treatments by Western blot analysis.

### 2.3 真核表达质粒转染对A549细胞生长的影响

真核表达质粒pVAX-iNOS分别转染A549细胞24 h、48 h和72 h后，通过MTT法对细胞的生长活力进行测定。结果显示，基因转染24 h时，细胞相对生长活力为89.83%，而转染48 h时，细胞生长活力明显受到抑制，达到约58.33%，而72 h时细胞生长活力约为47.88%（图 3，*P* < 0.01）。可以看出，基因转染48 h时，细胞的生长变化相对最为明显。

**3:**
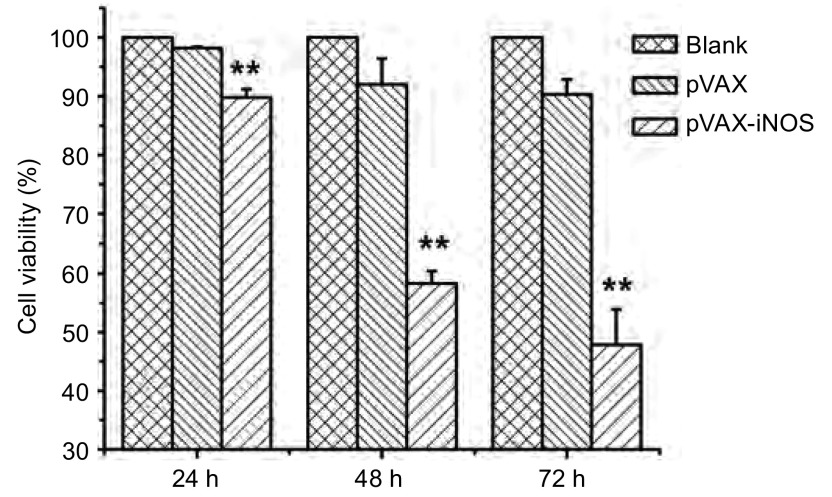
MTT法检测转染pVAX-iNOS基因对A549细胞生长的影响（^**^P < 0.01） Inhibition of cell proliferation of A549 cells after transfection of pVAX-iNOS for 24 h, 48 h and 72 h by MTT assay (^**^P < 0.01)

### 2.4 真核表达质粒转染对A549细胞凋亡的影响

将pVAX-iNOS质粒转染A549细胞，以未转染和仅转染空载体pVAX的细胞为阴性对照。转染48 h后观察到细胞生长受到抑制。Hoechst 3235染色后，经电子荧光显微镜下观察可见，经pVAX-iNOS转染的细胞在紫外光激发时发出明亮的蓝色荧光，核出现固缩裂解为碎块，产生明显的凋亡小体（图 4）；转染了空载体或未转染的细胞生长状态良好，几乎看不见凋亡小体。该结果表明pVAX-iNOS质粒在体外具有明显诱导肺癌细胞A549凋亡的作用。

**4:**
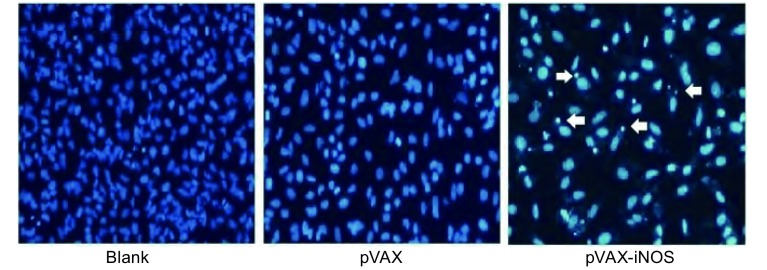
Hoechest 3235染色检测A549细胞转染pVAX-iNOS 48 h后的细胞凋亡形态 Induction of apoptosis of A549 cells transfected with pVAX-iNOS for 48 h by staining with Hoechst 3235

### 2.5 真核表达质粒转染对A549细胞迁移能力的影响

真核表达质粒pVAX-iNOS转染A549细胞，以未转染的A549细胞和仅转染空载体pVAX的细胞为阴性对照。转染24 h后，在各组单层细胞中间划出一道裸露无细胞带，取宽度相等的位置观察细胞迁移的情况。0 h时现各组细胞划痕边缘整齐，6 h、12 h和24 h后各组划痕边缘变得稍模糊，空白组及pVAX组的细胞向裸露的中间区域迁移，pVAX-iNOS组的细胞划痕边缘虽有模糊，但未见有细胞脱离，考虑为转染导致部分细胞坏死或凋亡所致。36 h后空白组及pVAX组的细胞继续向中间区域生长，48 h后可明显观察到空白组及pVAX组的划痕区域变窄，而pVAX-iNOS仅有少部分细胞向中间迁移生长，划痕区域清晰可见（图 5）。该结果表明pVAX-iNOS质粒在体外转染肺癌细胞A549具有明显抑制细胞发生迁移的作用。

**5:**
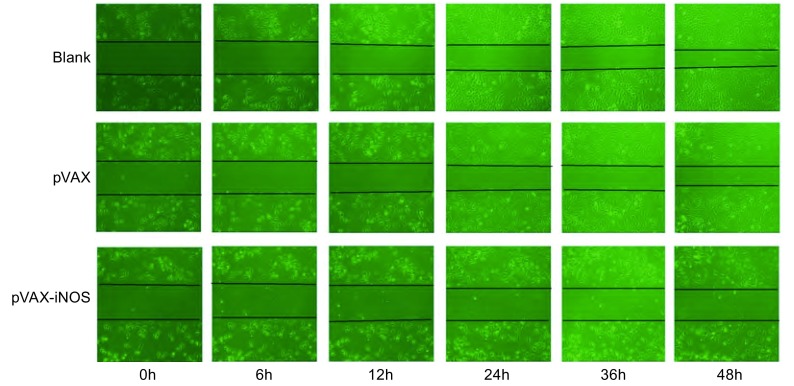
划痕实验检测pVAX-iNOS转染对A549细胞迁移能力的影响 Effect of the enhanced expression of iNOS on the migration ability of A549 cells transfected with pVAX-iNOS by a scratch assay

## 3 讨论

肺癌在全世界范围内是发病率和死亡率最高的恶性肿瘤。肺癌的发生和转移与多种基因的异常表达及相互作用密切相关。基因治疗已被认为是一种极具前景的肿瘤治疗方法。近年来，NO的双重生物学效应在肿瘤研究中得到了普遍重视，一方面，肿瘤细胞iNOS持续产生合适浓度NO可作为重要的信使分子，调节与细胞增殖相关基因的表达，增加肿瘤血供和血管形成，促进肿瘤增殖；另一方面，高浓度的NO对细胞又是一种损害，可通过细胞毒作用减缓肿瘤的生长和转移，抑制血管生成、促进分化和凋亡而起到抗肿瘤效应^[5-8]^，同时，研究^[7-12]^表明，高表达iNOS可抑制肝癌、恶性黑色素瘤、胰腺癌、乳腺癌等肿瘤的致瘤性和转移特性，并且还可提高结直肠癌^[23]^、卵巢癌^[24]^和非小细胞肺癌^[21]^等患者的存活率。因此，通过激发肿瘤细胞释放大量的NO可能是抗肿瘤治疗的一个新的方法^[13]^。

外源性诱导*iNOS*基因表达的方法包括NO供体药物、细胞因子和功能性*iNOS*基因的转染，但全身高浓度给予NO供体药物容易导致低血压的发生，细胞因子刺激法和NO供体法存在耐药性差、副反应大、特异性不强、毒性大等缺点，因此临床上应用存在一定困难^[18, 25]^，而*iNOS*基因转染可基本避免细胞因子刺激法和NO供体法的缺陷，目前已经在乳腺癌、结直肠癌、前列腺癌、卵巢癌、黑色素瘤、肾癌、甲状腺瘤等肿瘤中对iNOS转基因后产生的抗肿瘤效应进行了研究^[14-20]^。

Soler等^[14]^首次报道在甲状腺瘤模型中，iNOS可作为一个自杀基因进行治疗，并表现出一种强烈的抗肿瘤效应。尽管质粒的转染效率比较低，由于NO的弥散可导致强烈的旁观者效应，*iNOS*基因的抗肿瘤效应非常明显，且*iNOS*基因在肿瘤中可被可靠地定向到肿瘤细胞而避免产生全身毒性作用。同样，在黑色素瘤和肾癌细胞中也发现了类似的旁观者效应^[15]^。Jenkins等^[26]^将*iNOS*基因转染入结肠癌细胞后，持续分泌大量NO，并可抑制肿瘤细胞的增殖能力。Xie等^[27]^对荷瘤小鼠（K-1753细胞）给予*iNOS*基因治疗后，肿瘤出现了明显的生长抑制；体外实验也同样证实，通过转染*iNOS*基因可导致肿瘤细胞发生溶解和诱导旁细胞发生凋亡。Worthington等^[20]^通过使用辐射诱导型启动子（野生型激活片段1，WAF1）和阳离子脂质体将*iNOS*基因转染到肿瘤细胞后观察其对肿瘤细胞及其血管的影响，结果发现转染后可使鼠尾动脉扩张65%，从而增加传统的分次（割）放射治疗的潜在疗效；并且，转基因后还可延迟肿瘤的生长和促进肿瘤细胞发生凋亡。Adams等^[16]^通过将iNOS基因治疗和化疗药物顺铂联合对前列腺癌细胞株进行研究，观察iNOS转染后在大部分细胞中均显示了细胞毒作用，同时发现*iNOS*基因转染还可极大地提高顺铂的细胞毒效应。这些研究均发现，*iNOS*基因治疗在很大程度上能通过抑制肿瘤细胞生长、增殖、促进肿瘤细胞发生凋亡以及对周围肿瘤细胞产生旁观者效应等产生抗肿瘤效应。

在本研究中，我们通过高保真RT-PCR扩增iNOS目的片段，成功构建了真核表达质粒载体pVAX-iNOS。本研究选用的真核表达载体为pVAX，该质粒含有真核基因表达调控序列、CMV启动子有一个多克隆位点区，卡那霉素抗性和SV40加尾信号，在真核细胞内有高效表达活性，便于对质粒进行体外高效转染和体内治疗研究。通过阳离子脂质体转染的方法，本研究首次将pVAX-iNOS和pVAX转染A549肺癌细胞，在mRNA水平和蛋白水平检测到该基因表达上调；随后经MTT法对转染24 h、48 h和72 h的细胞进行生长活性检测，结果发现转基因可明显抑制A549肺癌细胞的增殖作用，尤以48 h相对抑制最为明显，考虑多为*iNOS*基因转染48 h后表达量最高，故其抗肿瘤作用亦最为明显；Hoechst染色和划痕试验发现，与空白组及空载体组相比，转染pVAX-iNOS质粒可明显诱导A549肺癌细胞发生凋亡及抑制细胞迁移。然而，肺癌的发生、发展是一个复杂的过程，涉及到多基因的异常作用以及各种其它因素的影响，因此通过基因治疗相互联合或基因治疗与化疗药物或放射治疗联合将极大地提高肿瘤基因治疗的效果。在下一步实验中，我们将联合化疗药物或放射治疗，并通过动物实验进一步研究pVAX-iNOS基因治疗对肺癌的抑制作用及抗肿瘤机制。本研究为将来肺癌基因治疗奠定了一定的研究基础，是临床肺癌基因治疗的一个新的探索。
